# Preoperative Serum Platelet-Lymphocyte Ratio as a Prognostic Factor in Cholangiocarcinoma Patients after Radical Resection: A Retrospective Analysis of 119 Patients

**DOI:** 10.1155/2019/8506967

**Published:** 2019-01-28

**Authors:** Ying Wu, Danyang Zhou, Guoping Zhang, Fengming Yi, Long Feng

**Affiliations:** ^1^Department of Oncology, The Second Affiliated Hospital of Nanchang University, 1 Minde Road, Nanchang, Jiangxi 330006, China; ^2^Jiangxi Key Laboratory of Clinical and Translational Cancer Research, Nanchang, Jiangxi 330006, China; ^3^Cancer Center, Union Hospital, Tongji Medical College, Huazhong University of Science and Technology, Wuhan 430022, China; ^4^Department of Oncology, Yuebei People's Hospital, Shaoguan, Guangdong 512000, China

## Abstract

**Aims:**

Although prognostic markers are important to establish therapeutic strategies in patients for conducting radical resection of cholangiocarcinoma (CCA), there is still a lack of simple, valid, and repeatable markers in clinical settings. We aim to evaluate the prognostic value of the preoperative serum platelet-lymphocyte ratio (PLR) in CCA patients who underwent radical resection.

**Methods:**

We retrospectively analyzed CCA patients who underwent radical resection surgery in our institution from January 2011 to June 2016. Baseline PLR and other clinical pathological data were measured when patients were diagnosed initially. The prognostic value of PLR in overall survival (OS) and progression-free survival (PFS) were analyzed with the Cox proportional hazard model and the Kaplan–Meier method.

**Results:**

This study retrospectively analyzed 119 patients who underwent radical resection of CCA. During a median follow-up time of 11.0 months, there were 99.2% recurrences and 42.9% who died, and the median OS and PFS were 9.4 months and 7.4 months, respectively. Multivariate Cox analysis identified that elevated levels of PLR (PLR > 157.25) as a significant factor predicted poorer OS (*P* = 0.018, HR: 2.160, 95% CI: 1.139-4.096) and PFS (*P* = 0.005, HR: 1.930, 95% CI: 1.220-3.053). In subgroup analysis, PLR also effectively predicted OS (*P* = 0.016, HR: 2.515, 95% CI: 1.143-5.532) and PFS (*P* = 0.042, HR: 1.908, 95% CI: 0.982-3.713) in CCA patients with positive lymphatic metastasis and/or positive surgical margin who required adjuvant therapy.

**Conclusions:**

The preoperative serum PLR is an independent prognostic factor for OS and PFS in CCA patients after radical resection, including patients requiring adjuvant therapy.

## 1. Introduction

Cholangiocarcinoma (CCA) is composed of mutated epithelial cells that originate from bile ducts [1]. CCA is the second most common primary liver malignancy which accounts for 5% to 30% of primary liver tumors [2]. The incidence is exceptionally high in developing countries, including Thailand, China, and South Korea [3]. According to their anatomical location, CCA is classified into intrahepatic CCA (iCC), perihilar CCA (pCC), and distal CCA (dCC), and the tumor location affects the pathogenesis and outcome [4].

Traditional radical resection is the only radical treatment for CCA patients. Unfortunately, owing to a majority of patients with advanced stages at diagnosis, the prognosis remains dismal [5, 6]. The overall R0 resection rate presents below 64.6% and 5-year overall survival below 20% [7, 8]. Additionally, adjuvant chemotherapy is recommended therapy for a patient with positive lymphatic metastasis and/or positive surgical margin after surgery according to the current guideline [9, 10]. However, there are still some CCA patients with the same stages for whom the standard of therapy and prognosis are significantly different in clinical practice [11, 12].

Etiologic and experimental evidence implicates that inflammation is a dominating factor in the pathogenesis and progression of CCA [13, 14]. Among inflammatory biomarkers, the high PLR, as a simple, effective, and quantifiable serum test, has been reported to have association with poorer prognoses in patients with various cancers [15–18]. Recent data reported that high PLR was predictive of poorer prognosis and early recurrence in iCC, pCC, and malignant obstructive jaundice (MOJ) [16, 19, 20]. However, the study on the prognosis of PLR for CCA patients after radical resection, especially patients requiring adjuvant therapy, is rare. Based on this background, we retrospectively analyzed the prognostic significance of the preoperative serum PLR for CCA patients who underwent radical resection to support clinical decision-making.

## 2. Methods

### 2.1. Patient

We retrospectively analyzed CCA patients who underwent radical resection surgery from an institutional database at the Department of Surgery, the Second Affiliated Hospital of Nanchang University, from January 2011 to June 2016. Pathological diagnosis of CCA was confirmed in all cases. Patients with nonoperatively treated CCA, distal metastasis and patients who underwent preoperative therapies (transcatheter arterial chemoembolization, radio-frequency ablation, percutaneous transhepatic catheter drainage, or neoadjuvant chemotherapy), pathologically confirmed mixed hepatocellular carcinoma, and patients who died of postoperative complications were excluded from the study. Clinical and hematologic examinations were measured at the time of diagnosis. Multidetector computed tomography (CT) and/or magnetic resonance imaging (MRI) were performed routinely to evaluate the local or distant extension of the primary tumors.

All patients underwent standard surgical treatment according to the recommended therapy [21, 22]. Resected specimens were histologically examined by specialized pathologists, and lymph node metastasis was evaluated in all specimens. Surgical margins were considered positive if infiltration of cancer cells was observed along the proximal, distal bile duct transection line or dissected peripancreatic soft-tissue margins. Tumor stage, lymph node metastasis, and final stage were classified based on the 7th edition of the International Union Against Cancer (UICC) tumor-node-metastasis (TNM) classification.

### 2.2. Follow-Up Strategy

All patients were followed regularly with CT and/or MRI every 3-6 months. Recurrence was defined as radiological evidence of intra-abdominal or abdominal soft tissue around the surgical site, or else distant metastasis. For patients who died, survival time after surgery and the result of death were recorded. For survivors (as of March 1, 2017), postsurgical time and recurrence status were recorded instead.

### 2.3. Statistical Analysis

PFS and OS were measured from the date of surgery to recurrence or death or last follow-up evaluation. The *χ*^2^ test or Fisher's exact probability was used for categorical variables. Univariate and multivariate analyses were documented using the Cox proportional hazard model, and all of the significant characteristics on univariate analysis were carried into multivariate analysis. Statistical analyses were performed using SPSS 18 for Windows (SPSS Inc., Chicago, IL, USA). All *P* values were two-sided, and *P* < 0.05 was considered significant.

## 3. Results

### 3.1. Clinical and Pathological Characteristics

We enrolled 119 patients with CCA who underwent surgical resection in the present study. The clinical and pathological characteristics of all the 119 patients are listed in Table 1. The best cutoff for preoperative serum PLR (PLR = 157.25) was determined from the median values and referred from previous literature reports [16, 19, 20]. On pathological analysis, the total R0 resection rate was 67.2% and the positive rate of lymphatic metastasis was 15.1%. The rate of R0 resection at iCC, pCC, and dCC was 64.5%, 55.6%, and 81.6%, respectively.

### 3.2. Follow-Up and Assessment of Prognosis

During a median follow-up time of 11.0 months, there were 99.2% (118/119) recurrences and 42.9% (51/119) who died, and the median OS and PFS were 9.4 months and 7.4 months, respectively. According to the best cutoff, the patients were divided into a low PLR group (*n* = 60) and a high PLR group (*n* = 59). The median OS and PFS of the low PLR group were 16.0 months and 11.0 months, respectively. These results were significantly more prolonged compared with the elevated PLR group whose median OS and PFS were 7.0 months and 5.0 months, respectively. In addition, the median PFS of iCC, pCC, and dCC was 5.0 months, 6.5 months, and 9.5 months, respectively, and OS of iCC, pCC, and dCC was 9.0 months, 9.5 months and 12.0 months, respectively.

### 3.3. Univariate and Multivariate Analyses of Prognostic Factors for Recurrence and Survival of CCA Patients

Overall demographics were similar, but patients with elevated preoperative serum PLR were correlated with female (*P* = 0.019) and positive surgical margin (*P* = 0.026) (Table 2).

**Figure 1:**
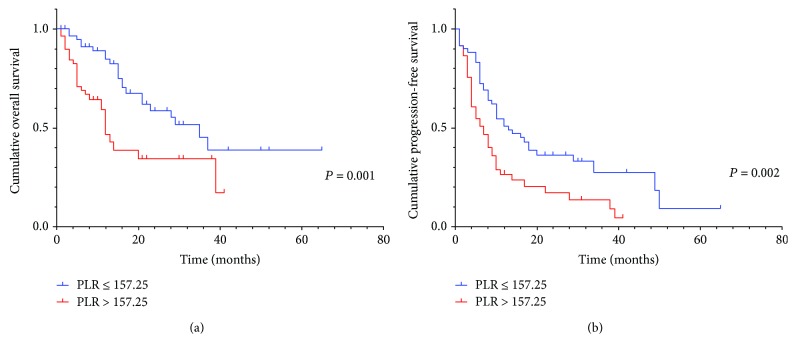
(a) Kaplan–Meier curve for OS of 119 CCA patients stratified by PLR. (b) Kaplan–Meier curve for PFS of 119 CCA patients stratified by PLR.

**Figure 2:**
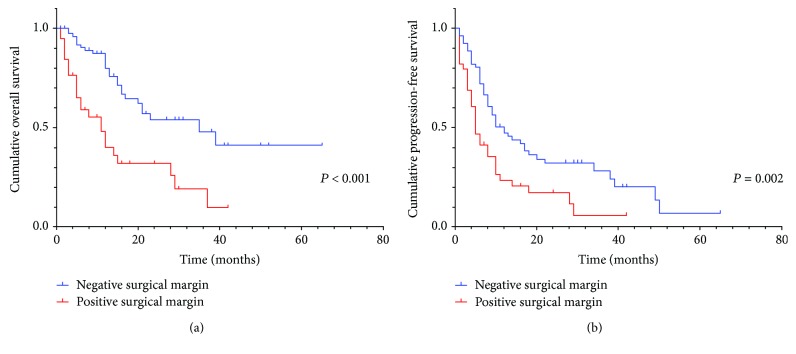
(a, b) Kaplan–Meier curve for OS and PFS of 119 CCA patients stratified by surgical margin.

Univariate analysis revealed that preoperative serum PLR (*P* = 0.001, HR: 2.493, 95% CI: 1.412-4.400) (Figure 1(a)), age, Eastern Cooperative Oncology Group (ECOG), surgical margin (*P* < 0.001, HR: 3.066, 95% CI: 1.762-5.332) (Figure 2(a)), differentiation, and CA199 were prognostic factors for OS. The result of univariate analysis was similar for PFS: preoperative serum PLR (*P* = 0.002, HR: 1.979, 95% CI: 1.269-3.086) (Figure 1(b)), tumor location, surgical margin (*P* = 0.002, HR: 1.979, 95% CI: 1.263-3.101) (Figure 2(b)), tumor size, and differentiation.

All of the factors with significant difference (*P* < 0.05) by univariate analysis were imported to multivariate analysis. Multivariate analysis demonstrated that preoperative serum PLR (*P* = 0.005, HR: 1.930, 95% CI: 1.220-3.053), differentiation (*P* < 0.001, HR: 0.411, 95% CI: 0.287-0.587), and tumor location (*P* < 0.001, HR: 0.566, 95% CI: 0.428-0.749) were independent risk factors for PFS (Table 3). PLR (*P* = 0.018, HR: 2.160, 95% CI: 1.139-4.096), differentiation (*P* < 0.001, HR: 0.388, 95% CI: 0.239-0.630), and CA199 (*P* = 0.001, HR: 3.689, 95% CI: 1.706-7.978) were independent risk factors for OS (Table 4).

**Figure 3:**
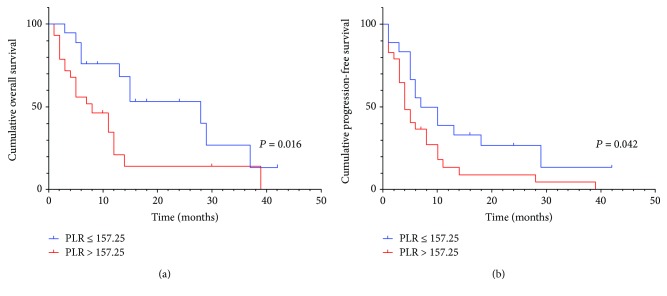
(a) Kaplan–Meier curve for OS and PFS of 47 CCA patients stratified by lymphatic metastasis and/or positive surgical margin. (b) Kaplan–Meier curve for PFS of 47 CCA patients stratified by lymphatic metastasis and/or positive surgical margin.

**Figure 4:**
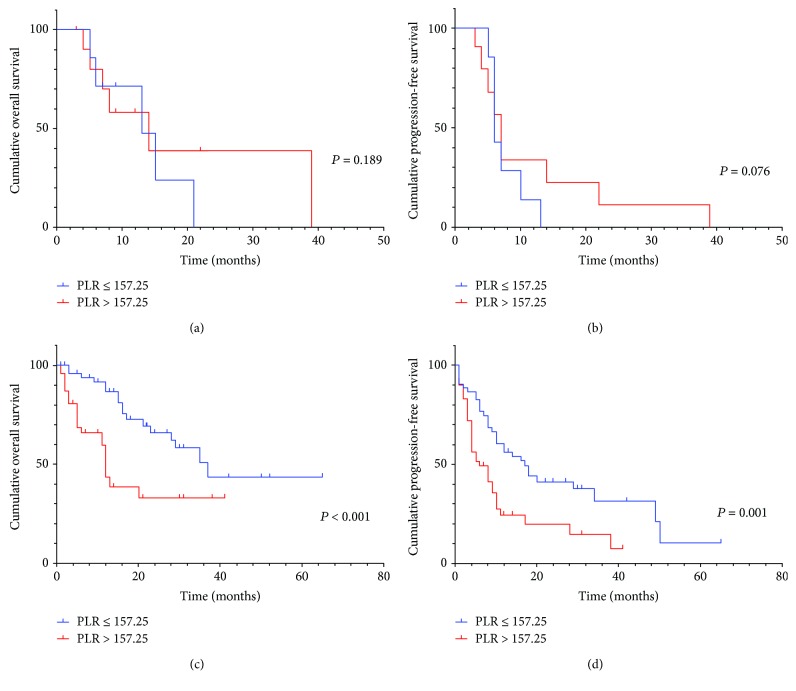
(a, b) Kaplan–Meier curve for OS and PFS of 18 positive lymphatic metastasis patients stratified by PLR. (c, d) Kaplan–Meier curve for OS and PFS of 101 negative lymphatic metastasis patients stratified by PLR.

**Figure 5:**
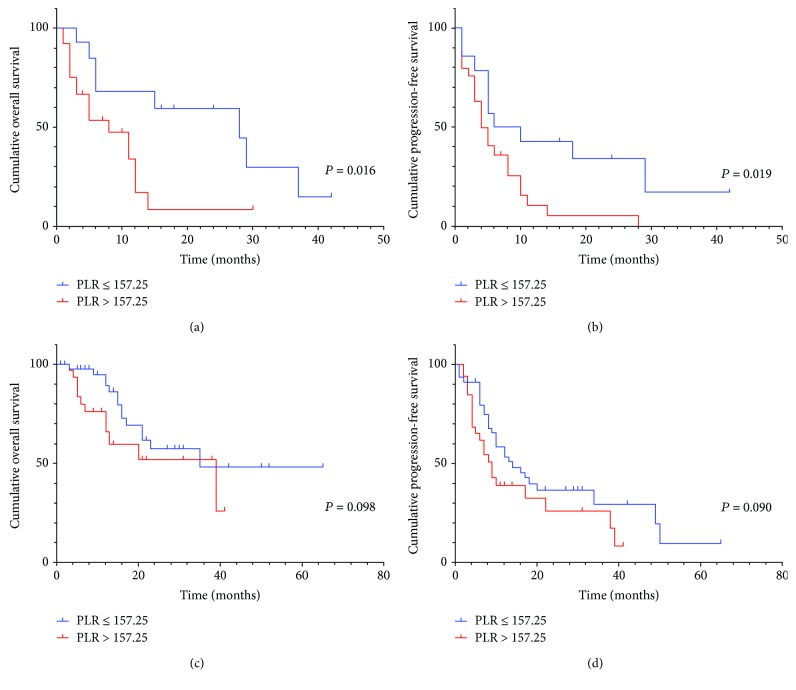
(a, b) Kaplan–Meier curve for OS and PFS of 39 positive surgical margin patients stratified by PLR. (c, d) Kaplan–Meier curve for OS and PFS of 80 negative surgical margin patients stratified by PLR.

In subgroup analysis, preoperative serum PLR was also significantly associated with OS (*P* = 0.016, HR: 2.515, 95% CI: 1.143-5.532) in patients with positive lymphatic metastasis and/or positive surgical margin (Figure 3(a)), but not statistically different from PFS (*P* = 0.042, HR: 1.908, 95% CI: 0.982-3.713) (Figure 3(b)). Besides, the preoperative serum PLR was significant with OS and PFS in patients with negative lymphatic metastasis (Figure 4(c) and 4(d)) and positive surgical margin (Figures 5(a) and 5(b)), but not in positive lymphatic metastasis (Figures 4(a) and 5(b)) and negative surgical margin (Figures 5(c) and 5(d)).

## 4. Discussion

There is increasing evidence that a negative resection can achieve better survival outcomes through radical resection, while others suggest that using even more aggressive resection to achieve negative margin is not a significant predictor of outcome [23–29]. Besides, previous studies also have demonstrated that the use of adjuvant therapy in high-risk patients could reduce recurrence and prolong survival after radical resection in patients with CCA [20, 30–36]. Therefore, this is a challenge to find a simple, effective, and reproducible clinical predictive factor that can initially determine the prognosis of patients with CCA after radical surgery, especially patients needing further treatment.

The tumor microenvironment largely orchestrated by inflammatory cells is an indispensable participant in the neoplastic process, fostering proliferation, survival, and migration [37, 38]. Increasing evidence elucidates that the elevations of systemic inflammatory markers including PLR are associated with poorer survival in patients with many cancers [39–43]. Although preoperative serum platelet and lymphocyte counts are the basic criteria for assessing the risk of surgery, only a few reports revealed the role of preoperative PLR in predicting the outcome in CCA patients who underwent radical resection. Analysis of Chen et al., Jin et al., and Saito et al. reported that elevated preoperative serum PLR predicts a poorer clinical prognosis in patients with iCC, pCC, and MOJ, respectively, but does not include all CCA types (including iCC, pCC, and dCC) [16, 19, 20].

Our results demonstrated that the elevated level of preoperative PLR (PLR > 157.25) as a significant factor predicted poor OS and PFS in CCA patients who underwent radical resection. Besides, preoperative serum PLR was more likely to increase in positive surgical margin patients with earlier recurrence and shorter survival. In addition, in previous studies, positive surgical margin was the high-risk factor for the prognosis of CCA patients who underwent radical resection [44]. We also demonstrated that tumor location was an independent prognostic factor affecting PFS but there was no significant difference in OS. Distal CCA patients have a significantly prolonged survival, which may be related to a high R0 resection rate.

On further analysis, the preoperative serum PLR was also significant with OS in patients with positive lymphatic metastasis and/or positive surgical margin with a poorer prognosis which require adjuvant therapy. Although PLR was not significantly associated with OS and PFS in lymph node-positive patients in the subgroup analysis, there was a tendency to separate between the K-M survival curves possibly due to a small sample size (*N* = 18).

As mentioned above, despite this study having many clinical implications, we should be clear that it is a retrospective study with its own limitations. First, our study was conducted in a single center, and only 119 patients were included in this study. The collection of multicenter data to expand the sample size is the next step that needs to be done. Prospective randomized, controlled studies have revealed that systemic chemotherapy extends survival in advanced-stage and postoperative patients [11, 45]. Due to some patients with positive lymph node metastasis and/or positive surgical margins who have not received chemotherapy (adjuvant chemotherapy/TACE) or lack of standardized circulating therapy, clinical outcomes are general poor. Finally, whether patients with a lower level of preoperative serum PLR benefit more from aggressive surgery needs to be verified by further clinical trials.

## 5. Conclusion

These results suggest that preoperative serum PLR not only is an independent prognostic factor for OS and PFS in patients with CCA who underwent radical resection but also predicts the prognosis of patients with positive lymphatic metastasis and/or positive surgical margin requiring adjuvant treatment. Depending on this, preoperative serum PLR is a simple and effective prognostic factor for patients undergoing radical resection of CCA, including patients requiring adjuvant therapy.

## Figures and Tables

**Table 1:** Patient demographics of CCA patients.

Variables	Median (interquartile range) or number (%)
Gender (female/male)	68 (57.1)/51 (42.9)
Age (years)	60 (33-80)
ECOG (=2/<2)	108 (90.8)/11 (9.2)
Tumor location (iCC/pCC/dCC)	45 (37.8)/36 (30.3)/38 (31.9)
Lymphatic metastasis (no/yes)	101 (84.9)/18 (15.1)
Surgical margin (negative/positive)	80 (67.2)/39 (32.8)
Tumor size (mm)	35 (10-120)
Differentiation (poor/moderated/well)	44 (37.0)/63 (52.9)/12 (10.1)
Vascular invasion (no/yes)	111 (93.3)/8 (6.7)
Nerve invasion (no/yes)	101 (84.9)/18 (15.1)
PLR	157.25 (49.4-623.1)
CA199 (U/ml)	161.88 (4.8-100.0)
TBIL (*μ*mol/L)	114.99 (5.2-536.9)

Abbreviations: PLR: platelet-lymphocyte ratio; ECOG: Eastern Cooperative Oncology Group; iCC: intrahepatic cholangiocarcinoma; pCC: perihilar cholangiocarcinoma; dCC: distal cholangiocarcinoma; CA199: carbohydrate antigen 19-9; TBIL: total bilirubin.

**Table 2:** Correlation between preoperative serum PLR and clinicopathological characteristics in CCA (*N* = 119).

Variable	*N* = 119	PLR		
		≤157.25 (*N* = 60)	>157.25 (*N* = 59)	*P* value
Gender				**0.019**
Female	68	28	40	
Male	51	32	19	
Age (years)				0.517
<60	63	30	33	
≥60	56	30	26	
ECOG				0.362
<2	11	4	7	
=2	108	56	52	
Tumor location				0.436
iCC	45	25	20	
pCC	36	15	21	
dCC	38	20	18	
Lymphatic metastasis				0.741
Negative	101	54	46	
Positive	18	6	13	
Surgical margin				**0.026**
Negative	80	46	34	
Positive	39	14	25	
Tumor size (mm)				0.143
≤40	78	46	38	
>40	41	14	21	
Differentiation				0.094
Poor	44	17	25	
Moderated	63	34	32	
Well	12	9	3	
Vascular invasion				0.980
No	111	56	55	
Yes	8	4	4	
Nerve invasion				0.636
No	101	50	51	
Yes	18	10	8	
CA199 (U/ml)				0.954
≤38	34	17	17	
>38	85	43	42	
TBIL (*μ*mol/L)				0.784
≤115	60	31	29	
>115	59	29	30	

Abbreviations: PLR: platelet-lymphocyte ratio; ECOG: Eastern Cooperative Oncology Group; iCC: intrahepatic cholangiocarcinoma; pCC: perihilar cholangiocarcinoma; dCC: distal cholangiocarcinoma; CA199: carbohydrate antigen 19-9; TBIL: total bilirubin; HR: hazard ratio; CI: confidence; *P* < 0.05 was considered significant.

**Table 3:** Kaplan–Meier method and Cox proportional hazard regression analysis of patients' overall survival.

Variable	Univariable		Multivariable	
	HR (95% CI)	*P* value	HR (95% CI)	*P* value
Gender (female/male)	2.273 (0.602-1.866)	0.793		
Age (years) (<60/≥60)	1.978 (1.118-3.498)	**0.015**	1.317 (0.619-2.799)	0.474
ECOG (<2/=2)	2.983 (1.372-6.484)	**0.003**	1.112 (0.762-1.622)	0.582
Tumor location (iCC/pCC/dCC)	0.327 (0.513-0.996)	0.071		
Lymphatic metastasis (no/yes)	1.888 (0.963-3.704)	0.056		
Surgical margin (negative/positive)	3.066 (1.762-5.332)	**<0.001**	3.101 (1.678-5.729)	**<0.001**
Tumor size (mm) (≤40/>40)	1.339 (0.762-2.352)	0.300		
Differentiation (poor/moderated/well)	0.250 (0.235-0.595)	**<0.001**	0.388 (0.239-0.630)	**<0.001**
Vascular invasion (no/yes)	1.009 (0.313-3.249)	0.988		
Nerve invasion (no/yes)	0.820 (0.368-1.824)	0.619		
PLR (<157.25/≥157.25)	2.493 (1.412-4.400)	**0.001**	2.160 (1.139-4.096)	**0.018**
CA199 (U/ml) (≤38/>38)	2.379 (1.536-4.897)	**0.014**	3.689 (1.706-7.978)	**0.001**
TBIL (*μ*mol/L) (≤115/>115)	0.978 (0.564-1.696)	0.936		

Abbreviations: PLR: platelet-lymphocyte ratio; ECOG: Eastern Cooperative Oncology Group; iCC: intrahepatic cholangiocarcinoma; pCC: perihilar cholangiocarcinoma; dCC: distal cholangiocarcinoma; CA199: carbohydrate antigen 19-9; TBIL: total bilirubin; HR: hazard ratio; CI: confidence; *P* < 0.05 was considered significant.

**Table 4:** Kaplan–Meier method and Cox proportional hazard regression analysis of patients' progression-free survival.

Variable	Univariable		Multivariable	
	HR (95% CI)	*P* value	HR (95% CI)	*P* value
Gender (female/male)	1.056 (0.683-1.633)	0.802		
Age (years) (<60/≥60)	1.068 (0.693-1.646)	0.758		
ECOG (<2/=2)	1.419 (0.680-2.965)	0.332		
Tumor location (iCC/pCC/dCC)	0.406 (0.519-0.873)	**0.004**	0.566 (0.428-0.749)	**<0.001**
Lymphatic metastasis (no/yes)	1.641 (0.945-2.849)	0.066		
Surgical margin (negative/positive)	1.979 (1.263-3.101)	**0.002**	1.567 (0.989-2.482)	0.056
Tumor size (mm) (≤40/>40)	1.692 (1.088-2.633)	**0.015**	0.935 (0.571-1.531)	0.790
Differentiation (poor/moderated/well)	0.330 (0.293-0.593)	**<0.001**	0.411 (0.287-0.587)	**<0.001**
Vascular invasion (no/yes)	1.151 (0.488-2.715)	0.741		
Nerve invasion (no/yes)	0.854 (0.461-1.582)	0.605		
PLR (<157.25/≥157.25)	1.979 (1.269-3.086)	**0.002**	1.930 (1.220-3.053)	**0.005**
CA199 (U/ml) (≤38/>38)	1.354 (0.833-2.199)	0.205		
TBIL (*μ*mol/L) (≤115/>115)	0.870 (0.565-1.340)	0.515		

Abbreviations: PLR: platelet-lymphocyte ratio; ECOG: Eastern Cooperative Oncology Group; iCC: intrahepatic cholangiocarcinoma; pCC: perihilar cholangiocarcinoma; dCC: distal cholangiocarcinoma; CA199: carbohydrate antigen 19-9; TBIL: total bilirubin; HR: hazard ratio; CI: confidence; *P* < 0.05 was considered significant.

## Data Availability

The datasets used or analyzed during the current study are available from the corresponding author on reasonable request.

## References

[1] Oliveira I. S., Kilcoyne A., Everett J. M., Mino-Kenudson M., Harisinghani M. G., Ganesan K. (2017). Cholangiocarcinoma: classification, diagnosis, staging, imaging features, and management. *Abdominal Radiology*.

[2] Ghouri Y. A., Mian I., Blechacz B. (2015). Cancer review: cholangiocarcinoma. *Journal of Carcinogenesis*.

[3] Global Burden of Disease Cancer Collaboration, Fitzmaurice C, Dicker D. (2015). The global burden of cancer 2013. *JAMA Oncology*.

[4] Banales J. M., Cardinale V., Carpino G. (2016). Expert consensus document: cholangiocarcinoma: current knowledge and future perspectives consensus statement from the European Network for the Study of Cholangiocarcinoma (ENS-CCA). *Nature Reviews Gastroenterology & Hepatology*.

[5] DeOliveira M. L., Kambakamba P., Clavien P. A. (2013). Advances in liver surgery for cholangiocarcinoma. *Current Opinion in Gastroenterology*.

[6] Doussot A., Lim C., Gómez-Gavara C. (2016). Multicentre study of the impact of morbidity on long-term survival following hepatectomy for intrahepatic cholangiocarcinoma. *British Journal of Surgery*.

[7] Wirth T. C., Vogel A. (2016). Surveillance in cholangiocellular carcinoma. *Best Practice & Research Clinical Gastroenterology*.

[8] de Jong M. C., Marques H., Clary B. M. (2012). The impact of portal vein resection on outcomes for hilar cholangiocarcinoma: a multi-institutional analysis of 305 cases. *Cancer*.

[9] Benson A. B., D'Angelica M. I., Abbott D. E. (2017). NCCN guidelines insights: hepatobiliary cancers, version 1.2017. *Journal of the National Comprehensive Cancer Network*.

[10] Valle J. W., Borbath I., Khan S. A. (2016). Biliary cancer: ESMO Clinical Practice Guidelines for diagnosis, treatment and follow-up. *Annals of Oncology*.

[11] Sharma A., Dwary A. D., Mohanti B. K. (2010). Best supportive care compared with chemotherapy for unresectable gall bladder cancer: a randomized controlled study. *Journal of Clinical Oncology*.

[12] Spolverato G., Kim Y., Ejaz A. (2015). Conditional probability of long-term survival after liver resection for intrahepatic cholangiocarcinoma: a multi-institutional analysis of 535 patients. *JAMA Surgery*.

[13] Rizvi S., Gores G. J. (2013). Pathogenesis, diagnosis, and management of cholangiocarcinoma. *Gastroenterology*.

[14] Coffelt S. B., de Visser K. E. (2014). Cancer: inflammation lights the way to metastasis. *Nature*.

[15] Hodek M., Sirák I., Ferko A. (2016). Neoadjuvant chemoradiotherapy of rectal carcinoma: baseline hematologic parameters influencing outcomes. *Strahlentherapie und Onkologie*.

[16] Jin H., Pang Q., Liu H. (2017). Prognostic value of inflammation-based markers in patients with recurrent malignant obstructive jaundice treated by reimplantation of biliary metal stents: a retrospective observational study. *Medicine*.

[17] Zhu Y., Si W., Sun Q., Qin B., Zhao W., Yang J. (2017). Platelet-lymphocyte ratio acts as an indicator of poor prognosis in patients with breast cancer. *Oncotarget*.

[18] Zhou X., Du Y., Huang Z. (2014). Prognostic value of PLR in various cancers: a meta-analysis. *PLoS One*.

[19] Chen Q., Dai Z., Yin D. (2015). Negative impact of preoperative platelet-lymphocyte ratio on outcome after hepatic resection for intrahepatic cholangiocarcinoma. *Medicine*.

[20] Saito H., Noji T., Okamura K., Tsuchikawa T., Shichinohe T., Hirano S. (2016). A new prognostic scoring system using factors available preoperatively to predict survival after operative resection of perihilar cholangiocarcinoma. *Surgery*.

[21] Fairweather M., Balachandran V. P., D’Angelica M. I. (2016). Surgical management of biliary tract cancers. *Chinese Clinical Oncology*.

[22] Hyder O., Marques H., Pulitano C. (2014). A nomogram to predict long-term survival after resection for intrahepatic cholangiocarcinoma: an Eastern and Western experience. *JAMA Surgery*.

[23] Tamandl D., Herberger B., Gruenberger B., Puhalla H., Klinger M., Gruenberger T. (2008). Influence of hepatic resection margin on recurrence and survival in intrahepatic cholangiocarcinoma. *Annals of Surgical Oncology*.

[24] Farges O., Fuks D., Boleslawski E. (2011). Influence of surgical margins on outcome in patients with intrahepatic cholangiocarcinoma: a multicenter study by the AFC-IHCC-2009 study group. *Annals of Surgery*.

[25] Washburn W. K., Lewis W. D., Jenkins R. L. (1995). Aggressive surgical resection for cholangiocarcinoma. *Archives of Surgery*.

[26] Shimizu H., Kimura F., Yoshidome H. (2010). Aggressive surgical resection for hilar cholangiocarcinoma of the left-side predominance: radicality and safety of left-sided hepatectomy. *Annals of Surgery*.

[27] Nakagohri T., Asano T., Kinoshita H. (2003). Aggressive surgical resection for hilar-invasive and peripheral intrahepatic cholangiocarcinoma. *World Journal of Surgery*.

[28] Lai E. C. H., Lau W. Y. (2005). Aggressive surgical resection for hilar cholangiocarcinoma. *ANZ Journal of Surgery*.

[29] Konstadoulakis M. M., Roayaie S., Gomatos I. P. (2008). Aggressive surgical resection for hilar cholangiocarcinoma: is it justified? Audit of a single center’s experience. *American Journal of Surgery*.

[30] Kim Y. S., Oh S. Y., Go S. I. (2017). The role of adjuvant therapy after R0 resection for patients with intrahepatic and perihilar cholangiocarcinomas. *Cancer Chemotherapy and Pharmacology*.

[31] Hester C., Nassour I., Adams-Huet B. (2018). Improved survival in surgically resected distal cholangiocarcinoma treated with adjuvant therapy: a propensity score matched analysis. *Journal of Gastrointestinal Surgery*.

[32] Nassour I., Mokdad A. A., Porembka M. R. (2018). Adjuvant therapy is associated with improved survival in resected perihilar cholangiocarcinoma: a propensity matched study. *Annals of Surgical Oncology*.

[33] Krasnick B. A., Jin L. X., Davidson J. T. (2018). Adjuvant therapy is associated with improved survival after curative resection for hilar cholangiocarcinoma: a multi-institution analysis from the U.S. extrahepatic biliary malignancy consortium. *Journal of Surgical Oncology*.

[34] Prabhu R. S., Hwang J. (2017). Adjuvant therapy in biliary tract and gall bladder carcinomas: a review. *Journal of Gastrointestinal Oncology*.

[35] Doherty M. K., Knox J. J. (2016). Adjuvant therapy for resected biliary tract cancer: a review. *Chinese Clinical Oncology*.

[36] Wirasorn K., Ngamprasertchai T., Khuntikeo N. (2013). Adjuvant chemotherapy in resectable cholangiocarcinoma patients. *Journal of Gastroenterology and Hepatology*.

[37] Candido J., Hagemann T. (2013). Cancer-related inflammation. *Journal of Clinical Immunology*.

[38] Grivennikov S. I., Greten F. R., Karin M. (2010). Immunity, inflammation, and cancer. *Cell*.

[39] Jagadesham V. P., Lagarde S. M., Immanuel A., Griffin S. M. (2017). Systemic inflammatory markers and outcome in patients with locally advanced adenocarcinoma of the oesophagus and gastro-oesophageal junction. *British Journal of Surgery*.

[40] Wariss B. R., de Souza Abrahao K., de Aguiar S. S., Bergmann A., Thuler L. C. S. (2017). Effectiveness of four inflammatory markers in predicting prognosis in 2374 women with breast cancer. *Maturitas*.

[41] Meaney C. L., Zingone A., Brown D., Yu Y., Cao L., Ryan B. M. (2017). Identification of serum inflammatory markers as classifiers of lung cancer mortality for stage I adenocarcinoma. *Oncotarget*.

[42] Ghuman S., Van Hemelrijck M., Garmo H. (2017). Serum inflammatory markers and colorectal cancer risk and survival. *British Journal of Cancer*.

[43] Fogelman D. R., Morris J., Xiao L. (2017). A predictive model of inflammatory markers and patient-reported symptoms for cachexia in newly diagnosed pancreatic cancer patients. *Supportive Care in Cancer*.

[44] DeOliveira M. L., Cunningham S. C., Cameron J. L. (2007). Cholangiocarcinoma: thirty-one-year experience with 564 patients at a single institution. *Annals of Surgery*.

[45] Stein A., Arnold D., Bridgewater J. (2015). Adjuvant chemotherapy with gemcitabine and cisplatin compared to observation after curative intent resection of cholangiocarcinoma and muscle invasive gallbladder carcinoma (ACTICCA-1 trial) - a randomized, multidisciplinary, multinational phase III trial. *BMC Cancer*.

